# Acute kidney injury in patients with SARS-CoV-2 infection

**DOI:** 10.1186/s13613-020-00734-z

**Published:** 2020-09-03

**Authors:** Adrien Joseph, Lara Zafrani, Asma Mabrouki, Elie Azoulay, Michael Darmon

**Affiliations:** 1 Service de médecine Intensive et de réanimation médicale, Hôpital Saint-Louis, Assistance-Publique Hôpitaux de Paris, Paris University, 1 avenue Claude Vellefaux, 75010 Paris, France; 2 ECSTRA Team, UMR 1153, Center of Epidemiology and Biostatistics, INSERM, Université de Paris, Paris, France; 3 INSERM U976, Université de Paris, Paris, France

**Keywords:** Acute kidney injury, COVID-19, Complement system proteins, Interleukin-6, Outcome, Intensive care units

## Abstract

**Background:**

Acute Kidney Injury (AKI) is a frequent complication of severe SARS-CoV-2 infection. Multiple mechanisms are involved in COVID-19-associated AKI, from direct viral infection and secondary inflammation to complement activation and microthrombosis. However, data are limited in critically-ill patients. In this study, we sought to describe the prevalence, risk factors and prognostic impact of AKI in this setting.

**Methods:**

Retrospective monocenter study including adult patients with laboratory confirmed SARS-CoV-2 infection admitted to the ICU of our university Hospital. AKI was defined according to both urinary output and creatinine KDIGO criteria.

**Results:**

Overall, 100 COVID-19 patients were admitted. AKI occurred in 81 patients (81%), including 44, 10 and 27 patients with AKI stage 1, 2 and 3 respectively. The severity of AKI was associated with mortality at day 28 (*p* = 0.013). Before adjustment, the third fraction of complement (C3), interleukin-6 (IL-6) and ferritin levels were higher in AKI patients. After adjustment for confounders, both severity (modified SOFA score per point) and AKI were associated with outcome. When forced in the final model, C3 (OR per log 0.25; 95% CI 0.01–4.66), IL-6 (OR per log 0.83; 95% CI 0.51–1.34), or ferritin (OR per log 1.63; 95% CI 0.84–3.32) were not associated with AKI and did not change the model.

**Conclusion:**

In conclusion, we did not find any association between complement activation or inflammatory markers and AKI. Proportion of patients with AKI during severe SARS-CoV-2 infection is higher than previously reported and associated with outcome.

## Background

Since December 2019, severe acute respiratory coronavirus 2 (SARS-CoV-2) has spread worldwide, causing more than 6.6 million cases and 390 000 deaths [1]. This pandemic has put unprecedented pressure on healthcare systems and especially on intensive care units (ICUs).

Acute Kidney Injury (AKI) is a frequent complication of severe SARS-CoV-2 infection but data are scarce in ICUs. AKI has been previously reported with an average incidence of 11% (8–17%) overall, with highest ranges in the critically ill (23%; 14–35%) [2–4]. Different applications of the Kidney Disease Improving Global Outcomes (KDIGO) criteria for AKI, in particular different methods to estimate missing baseline creatinine and handling urinary output, can cause important variations of estimated incidence [5, 6] and may contribute to the discrepancies among these studies.

Multiple mechanisms are involved in COVID-19-associated AKI, ranging from direct viral infection of the kidney and secondary inflammation to complement activation and microthrombosis [7]. In particular, severe COVID-19 is associated with uncontrolled systemic inflammatory response with high levels of IL-6 [8] that could potentially lead to intrarenal inflammation and increased vascular permeability and share several features with hyperferritinemic syndromes such as macrophage activation syndrome [9]. Furthermore, unrestrained activation of complement leads to endothelial cell dysfunction and intravascular coagulation that could participate in COVID-19-associated AKI [10]. Both IL-6 [11] and complement [12] have been proposed as therapeutic targets and understanding their role in COVID-19-associated AKI is therefore a priority.

However, most of the studies performed to date gave little data regarding definition of AKI or influence of inflammation and complement markers on AKI.

In this study, we sought to describe the prevalence, risk factors and prognostic impact of AKI during COVID-19 in the ICU.

## Methods

### Study design and cohort

We conducted a retrospective monocenter study including adult patients with laboratory confirmed SARS-CoV-2 infection admitted to the ICU of our university Hospital. All adult patients (age ≥ 18 years) who tested positive by polymerase chain reaction testing of a nasopharyngeal sample for COVID-19 and were hospitalized from March 1, 2020 to June 1, 2020 were eligible.

This study was approved by an institutional review board (French Intensive Care Society—CE SRLF n°20–32). Need for informed consent was waived as regard to the study observational design and in accordance with the French law. This study was conducted in accordance with the principles of the Declaration of Helsinki.

### Data collection, definitions and measurements

All data were obtained from medical records and patients’ charts. Baseline patients’ characteristics were collected, including demographics and comorbidities before ICU admission. The variables recorded regarding ICU admission and treatments were relative to clinical presentation, reason for ICU admission, diagnosis, therapies implemented and outcomes.

Blood sampling and routine biological testing were performed on the day of admission according to the standard laboratory protocols.

All samples were immediately centrifuged at 3000 rpm at 4 °C for 10 min, separated from the cells and stored at −80 °C until biochemical assays of complement proteins C3, C4 (nephelometry) and sC5B9 (ELISA), IL-6 (ELISA) were performed. The primary outcome was mortality at day 28.

### Definition of AKI

AKI was defined according to both urinary output and serum creatinine KDIGO criteria [13] as follows: stage 1—increase in serum creatinine by 0.3 mg/dl within 48 h or a 1.5–1.9 times increase in serum creatinine from baseline or urinary output < 0.5 ml/kg/h for 6–12 h within 7 days; stage 2—2.9 times increase in serum creatinine or urinary output < 0.5 ml/kg/h for ≥ 12 h within 7 days; stage 3—3 times or more increase in serum creatinine or to ≥ 4.0 mg/dl or initiation of RRT or urinary output < 0.3 ml/kg/h for ≥ 24 h or anuria for ≥ 12 h within 7 days. Patients were stratified according to the highest AKI stage attained during the first 7 days of ICU stay.

Baseline creatinine was defined as the best value in the 3 preceding months or if unavailable as the lowest value during ICU stay or was back calculated based on a glomerular filtration rate of 60 mL/min/1.73m^2^ with MDRD equation in patients without known chronic kidney disease. Chronic kidney disease (CKD) was defined according to the KDIGO definition.

Modified SOFA was defined as SOFA score [14] excluding the renal component.

### Statistical analysis

Continuous variables were described as median (interquartile range [IQR]) and compared between groups using the non-parametric Wilcoxon rank-sum test. Categorical variables were described as frequency (percentages) and compared between groups using Fisher’s exact test. Mortality was assessed using survival analysis.

Independent risk factors of day 28 mortality were assessed using Cox model. Conditional stepwise variable selection was performed with 0.2 as the critical *p*-value for entry into the model, and 0.1 as the *p*-value for removal. Interactions and correlations between the explanatory variables were carefully checked. Validity of proportional hazards assumption, influence of outliers, and linearity in relationship between the log hazard and the covariates were carefully checked.

Independent risk factors of AKI were assessed using logistic regression. Conditional backward stepwise variable selection was performed with 0.2 as the critical *p*-value for entry into the model, and 0.1 as the *p*-value for removal. Interactions and correlations between the explanatory variables were carefully checked. Influence of outliers, and linearity in relationship between the log hazard and the covariates were carefully checked. It was preplanned to force, one by one, in the final model inflammation biomarker, ferritin, complement pathway dosage and PEEP level at admission should these variable not be selected.

Kaplan–Meier graphs were used to express the probability of death from inclusion to day 28. Comparisons were performed using the log-rank test.

Overall, rate of missing data was 6.6% with rate of missing data among major outcome or covariates was < 5%. As regard to completeness of the dataset, no imputation of missing data was performed.

Statistical analyses were performed with R statistical software, version 3.6.2 (available online at https://www.r-project.org/) and the ‘Survival’ package was used. A *p* value < 0.05 was considered significant.

## Results

### Patients’ characteristics

Overall, 100 COVID-19 patients were admitted in our ICU between March and May 2020 and included in this study. Patients’ characteristics are described in Table 1. Median age was 59 years [53–67] and 70 patients (70%) were of male gender. Only 15 patients (15%) had no underlying disease. Hypertension (*n* = 56, 56%), diabetes (*n* = 30, 30%) and chronic kidney disease (*n* = 29, 29%) were the main comorbidities. Thirty-six patients were obese (*n* = 21, 21%) or overweight (*n* = 25, 25%) and 30 (30%) were treated with angiotensin converting enzyme inhibitors or angiotensin-receptor blockers. Median SOFA score at admission was 4 [2–7]. Median modified SOFA score [without the renal component] was 3 [2–7]. Fifty-six patients (55%) required mechanical ventilation and 51 (51%) vasopressor therapy.

**Table 1:** Patients’ characteristics according to AKI

	Overall	No AKI	AKI	*p* value
	*n* = 100	*n* = 19	*n* = 81	
Age (year)	59 [53–67]	54 [45–61]	60 [54–68]	0.05
Male gender	70 (70%)	11 (58%)	59 (73%)	0.32
Absence of underlying comorbidity	15 (15%)	6 (32%)	9 (11%)	0.06
Chronic Obstructive Pulmonary Disease	2 (2%)	0 (0)	2 (3%)	1.00
Asthma	8 (8%)	2 (11%)	6 (8%)	1.00
History of hypertension	56 (56%)	8 (42%)	48 (60%)	0.25
Diabetes	30 (30%)	3 (16%)	27 (34%)	0.21
Immunocompromized	26 (26%)	4 (21%)	22 (28%)	0.78
Heart failure	15 (15%)	0 (0)	15 (19%)	0.09
Chronic kidney disease	29 (29%)	2 (11%)	27 (33%)	0.09
Body Mass Index (Kg/m^2^)	28 [24–31]	26 [23–31]	28 [24–31]	0.48
*Obesity and overweight*				0.37
Overweight	25 (25%)	3 (16%)	22 (27%)	
Obese	21 (21%)	3 (16%)	18 (22%)	
Other	54 (54%)	13 (68%)	41 (51%)	
Chronic use of ACE/ARB	30 (30%)	3 (16%)	27 (33%)	0.22
Baseline serum creatinine (µmol/L)	65 [50–93]	63 [48–70]	67 [50–100]	0.22
SOFA score	4 [2–7]	2 [2, 3]	5 [2–7]	0.003
Nonsteroidal anti-inflammatory drugs (%)	1 (1%)	0 (0)	1 (1%)	1.00
Invasive mechanical ventilation	55 (55%)	6 (32%)	49 (61%)	0.04
PEEP at day 1	0 [0–10]	0 [0–4]	8 [0–10]	0.04
Renal replacement therapy	13 (13%)	0 (0)	13 (16%)	0.14
Vasopressors	51 (51%)	7 (37%)	44 (55%)	0.24
IL6 at day 0 (ng/mL)	118 [67–287]	136 [63–292]	113 [69–287]	0.98
C3 at day 0 (ng/mL)	1305 [1173–1550]	1495 [1285, 1630]	1260 [1160–1543]	0.06
C4 at day 0 (ng/mL)	348 [275–418]	351 [282–493]	348 [272–416]	0.69
sC5b9 at day 0 (ng/mL)	373 [270–471]	425 [317–516]	363 [266–450]	0.22
Ferritin at day 0 (mg/L)	1272 [636–2234]	1182 [495–1584]	1311 [695–2322]	0.12
Fibrinogen at day 0 (g/L)	6.8 [5.8–7.8]	7.2 [5.4–7.7]	6.7 [5.8–7.8]	0.94
Acute kidney injury -	81 (81%)	0 (0)	81 (100%)	< 0.001
*KDIGO stage*				< 0.001
No AKI	19 (19%)	19 (100%)	0 (0)	
Stage 1	44 (44%)	0 (0)	44 (54%)	
Stage 2	10 (10%)	0 (0)	10 (12%)	
Stage 3	27 (27%)	0 (0)	27 (33%)	
*KDIGO criteria fulfilled*				< 0.001
Creatinine criteria alone	28 (28%)	0 (0)	28 (35%)	
Oliguria alone	33 (33%)	0 (0)	33 (41%)	
Both criteria	20 (20%)	0 (0)	20 (25%)	
None	19 (19%)	19 (100%)	0 (0)	
Day-28 mortality	29 (29%)	1 (5%)	28 (35%)	0.02
*Specific treatment during ICU stay*				0.35
Chloroquine or Hydroxychloroquine	3 (3%)	0 (0)	3 (4%)	
Eculizumab	2 (2%)	0 (0)	2 (3%)	
Lopinavir/ritonavir	10 (10%)	0 (0)	10 (12%)	
Tocilizumab	1 (1%)	0 (0)	1 (1%)	
None	84 (84%)	19 (100%)	65 (80%)	

Results are presented as median (interquartile) or n (%)

*ACE* angiotensin converting enzyme inhibitors, *ARB* angiotensin receptor blockers

### Risk factors of AKI in COVID-19 patients

Rate of patients with missing baseline serum creatinine was 67% (*n* = 67) and did not differ between AKI and patients without AKI (74 vs. 65% respectively; *p* = 0.67).

AKI occurred in 81 patients (81%), including 44 patients, 10 patients, 27 patients with AKI stage 1, 2 and 3 respectively. Among patients with AKI, 33 (41%) met only urinary output KDIGO criteria, 28 (35%) met only creatinine criteria and 20 (25%) met both. Urinary output criteria alone was more frequently involved in diagnosis of milder stage of AKI (AKI stage 1 (*n* = 20, 45%) and stage 2 (*n* = 6, 60%) compared to 26% in stage 3 (*n* = 7, *p* = 0.007). Thirteen (13%) required renal replacement therapy during the first 7 days in ICU.

**Fig. 1:**
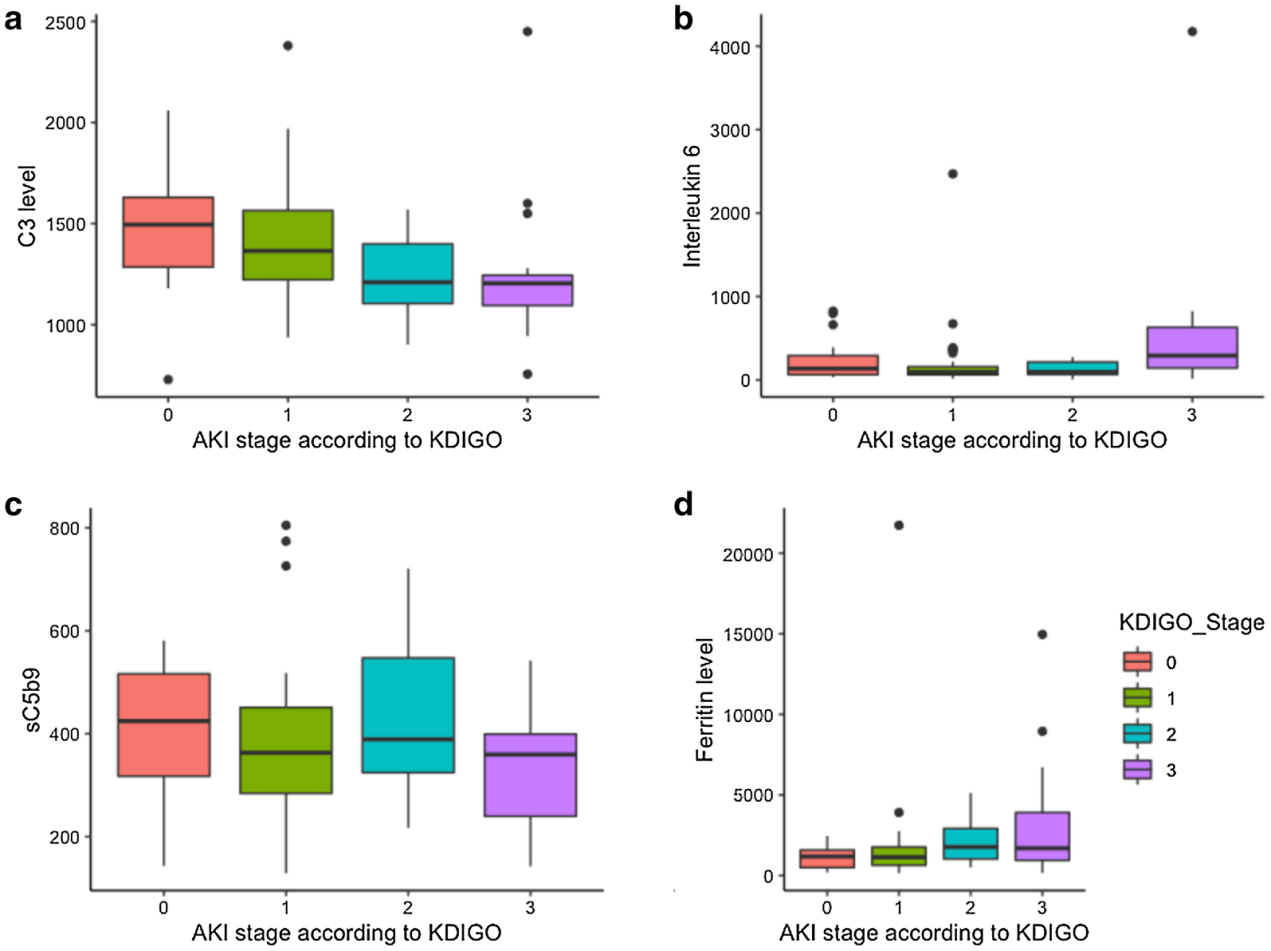
Boxplots depicting relationship between AKI severity and C3 [ng/mL] (*p* = 0.01) (**a**), IL-6 [ng/mL] (*p* = 0.02) (**b**), sC5b9 [ng/mL] (*p* = 0.42) (**c**) and ferritin levels [mg/L] (*p* = 0.03) (**d**) levels

Before adjustment, C3 (*p* = 0.01), IL-6 (*p* = 0.03) and ferritin levels (*p* = 0.03) were associated with AKI severity, whereas soluble C5b9 fraction was not different (*p* = 0.42) (Fig. 1)*.*

In a multivariate model incorporating baseline chronic kidney disease, only modified SOFA was significantly associated with the development of AKI (OR per point 1.29; 95% CI 1.04–1.70).

When forced in the final model, C3 (OR per log 0.25; 95% CI 0.01–4.66), IL-6 (OR per log 0.83; 95% CI 0.51–1.34), ferritin (OR per log 1.63; 95% CI 0.84–3.32), or PEEP level (OR per mmHg 1.04; 95% CI 0.91–1.22) were not associated with AKI and did not change the model.

However, after adjustment for modified SOFA and CKD, C3 value higher than median was significantly associated with a lower risk for AKI stage 2 or 3, compared to no AKI or AKI stage 1 (OR 0.17 95% CI [0.05–0.54], *p* = 0.004), while the association between AKI stage 2 or 3 and IL-6, Ferritin, sC5b9 and PEEP levels did not reach statistical significance (Additional file 1: Table S1 and Figure S1).

### Outcome analysis

**Fig. 2:**
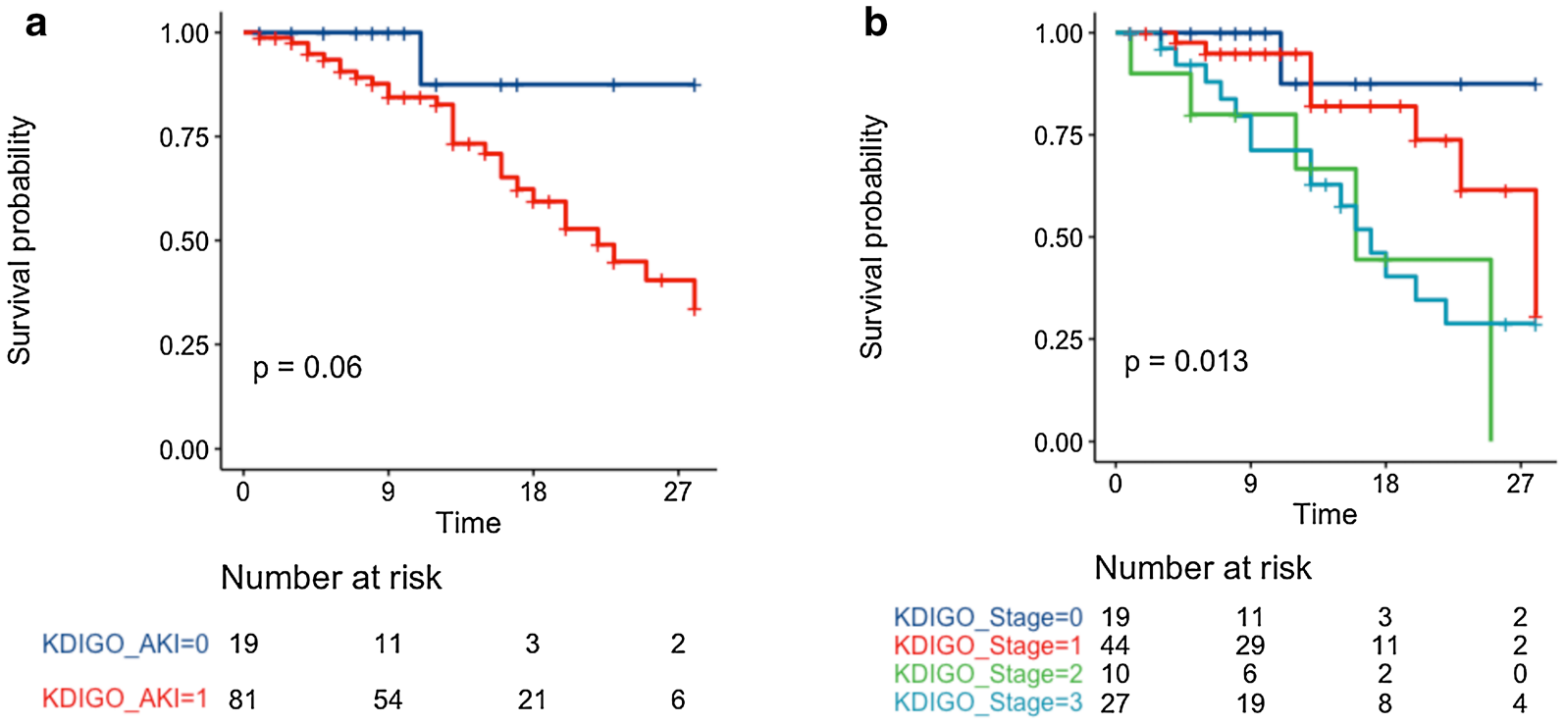
Kaplan–Meier curves for day-28 survival in patients with (*n* = 81) and without AKI (*n* = 19) (**a**) and with AKI stage 1 (*n* = 44), 2 (*n* = 10) and 3 (*n* = 27) (**b**)

Twenty-nine (29%) patients had died by day 28, 28 (35%) patients with AKI and 1 (5%) patient without AKI (*p* = 0.02). More than half of the patients with AKI stage 2 (*n* = 5, 50%) and 3 (*n* = 15, 56%) died before day 28. The severity of AKI was associated with mortality at day 28 (*p* = 0.013) (Fig. 2 and Additional file 1: Figure S2).

Patients who died were older (66 versus 57 years, *p* = 0.001), suffered more frequently from chronic kidney disease (52 versus 20%, *p* = 0.003), had higher SOFA score (8 versus 2, *p* < 0.001), ferritin levels (1805 versus 1148 mg/L, *p* = 0.004) and IL-6 levels (204 versus 103 ng/mL, *p* = 0.02), and were less often immunocompetent (46 versus 18%, *p* = 0.01). Neither comorbidities nor BMI were associated with death at day 28. Patients who died more often required mechanical ventilation (90 versus 41%, *p* < 0.001), vasopressors (90 versus 36%, *p* < 0.001) and renal replacement therapy (31 versus 6%, *p* = 0.002).

After adjustment for confounders, both AKI and severity (modified SOFA score per point) were associated with survival (Table 2).

**Table 2:** Factors associated with survival censored at day 28 (Cox Model) and risk of AKI (logistic regression)

	Estimate	95% CI	*p* value
Model 1 (survival censored at day 28)	Hazard ratio		
Modified SOFA score at admission (per point)	1.23	1.04–1.45	0.01
Acute Kidney Injury	3.08	1.12–8.45	0.03
Model 2 (acute kidney injury)	Odds ratio		
Chronic kidney disease	3.91	0.97–26.20	0.09
Modified SOFA score at admission (per point)	1.29	1.04–1.70	0.04

Model selection was performed according to forward variable selection conditioned on *p* value (critical entry *p* value < 0.2, critical exit *p* value < 0.1)

There was a similar trend towards poorer survival in patients with (*n* = 33) and without (*n* = 67) baseline serum creatinine (Additional file 1: Figure S3).

We also tested the interaction between missing baseline serum creatinine and reported results and did not find any significant interaction (Additional file 1: Table S2).

Treatments with lopinavir/ritonavir (*n* = 10), tocilizumab (*n* = 1), eculizumab (*n* = 2) or chloroquine (*n* = 3) were associated neither with mortality nor with the development of AKI.

## Discussion

In this study, we describe the incidence of AKI in 100 COVID-19 patients admitted to the ICU, and the link between AKI, inflammation markers and complement levels. The main results of this case series are the higher than previously reported incidence of AKI and its lack of association with IL-6, ferritin or complement factors C3 and sC5B9. The importance of AKI in COVID-19 patients has been increasingly recognized. If initial reports from China reported rates of AKI as low as 2.9–8% in severe patients [2, 15, 16], incidences from later studies ranged between 15% [17] and 44% [18] in critically ill patients. Most reports lack a clear operational AKI definition [19–21], but even in studies that used KDIGO serum creatinine criteria, diuresis was inconsistently taken into account, and none of them reported AKI stages. Our study is the largest published to date reporting incidence of AKI specifically in critically ill patients. With 81% of patients diagnosed with AKI, the rate of AKI in our study is much higher than previously reported.

Interestingly, one of the largest cohort from the United States reported a prevalence of AKI in COVID-19 patients requiring mechanical ventilation of 80% [22], close to our finding of 90%.

One strength of our study is the rigorous use of KDIGO criteria, including urinary output. Furthermore, given that many patients will present with AKI without a reliable baseline serum creatinine on record, estimation of the latter is of tremendous importance. Several methods to estimate missing baseline creatinine can lead to variations in incidence of AKI up to 15% [6]. Using complete KDIGO definition we found a 80% incidence of AKI during the first 7 days of ICU stay and a 90% incidence in patients requiring mechanical ventilation.

We also tested the hypotheses that COVID-19-associated-AKI is linked to the elevation of cytokines induced by SARS-CoV-2 infection [23], complement dysregulation [12] induced by SARS-CoV-2 infection or mechanical ventilation settings. High levels of IL-6 have been associated with the development of severe disease [24, 25] and acute respiratory distress syndrome [8] during COVID-19 infection, but the role of inflammation markers in COVID-19-induced-AKI remains speculative [7]. The deleterious role of IL-6 has been demonstrated in different models of AKI, including ischemic AKI, nephrotoxin-induced AKI and sepsis-induced AKI [26, 27]. In our study, IL-6 and ferritin levels correlated with severity but were not independently associated with AKI. The complement system represents the first response of the host immune system. It participates in the development of AKI [28] and has also been suspected to play a role in AKI in the context of SARS-CoV-2 infection [10, 12]. Recent studies showed a strong immunohistochemical staining of complement cascade components in the lungs [29] and kidneys [30] of severe COVID-19 patients. However, even if C3 levels were associated with AKI severity in univariate analysis, the association did not persist when forced into a multivariate model, and soluble C5b9 showed no association with AKI. C3 level was only independently associated with AKI stage 2 and 3 compared to no AKI or AKI stage 1. Overall, our results do not support a role for complement dysregulation in COVID-19-induced-AKI, even though complement dysregulation may be involved in the most severe forms of AKI.

Although our study did not include extensive coagulation explorations [31], the lack of difference in fibrinogen levels in patients with AKI when compared to patients without AKI does not support the evidence of a role of hypercoagulability in COVID-19-induced-AKI, suggested by the presence of thrombi in glomerular loops described by others [32, 33]. Last, high levels of PEEP in COVID-19 patients [34, 35] have also been suggested as a potential factor for increased AKI in severe COVID-19 patients [36]. In our study, PEEP levels were not independently associated with the development of AKI, but the lack of statistical power and longitudinal data on PEEP levels does not allow for any definite conclusion.

Our study also has limitations. First, despite being the largest focusing on COVID-19-induced-AKI in the ICU, the limited number of patients can translate into a lack of statistical power. Nevertheless, rate of AKI and sample size allow confirming a high AKI incidence with a reasonable level of confidence (81%; 95% CI 72–89). In addition, the monocenter design may have limited external validity of our findings. The proportion of missing baseline serum creatinine value (67%) may also be an issue. Missing baseline creatinine value is a common problem in research focusing on AKI, and although most studies do not report the proportion of missing baseline values, rates as high as 85% are common [22]. We provide sensitivity analyses (Additional file 1: Figure S3 and Table S2) to show that back calculation of missing baseline creatinine values unlikely results in a significant bias. Last, lack of association between inflammation biomarker, IL-6 or complement component do not preclude participation of these mechanisms to AKI. They only underline that in this setting and when adjusted to severity, these factors have little influence on AKI rate. Additional studies, assessing levels of proteinuria and hematuria [22, 37], pathological findings and translational research are needed to further explore different mechanisms that may participate to AKI during severe SARS-CoV-2 infection.

In conclusion, we did not find any association between complement activation or inflammatory markers and AKI. Our study suggests a tremendously high incidence of AKI in our cohort of critically ill COVID-19 patients, along with an independent association between AKI and outcome. Because of its marked influence on outcome, AKI should be identified promptly during SARS-CoV-2 infection.

## Supplementary information

**Additional file 1: Figure S1.** Boxplots depicting relationship between AKI stage 2 and 3 and C3 [ng/mL] (A), IL-6 [ng/mL] (B), sC5b9 [ng/mL] (C) and ferritin levels [mg/L] (D) levels, and predicted probabilities of severe AKI according to ferritin (E) and C3 (F) deciles (per log).** Figure S2.** Kaplan-Meier curve for day-28 survival in patients without AKI or with AKI stage 1 (n=63) compared to AKI stage 2 or 3 (n= 37).** Figure S3.** Kaplan-Meier curves for day-28 survival according to AKI (A and B) and AKI stages (C and D) in patients with (n=33, B and D) and without (n= 67, A and C) baseline creatinine value. **Table S1.** Factors associated with risk of AKI stage 2 and 3, compared to no AKI or AKI stage 1, after adjustment for modified SOFA and chronic kidney disease (logistic regression).** Table S2.** Interaction between missing baseline serum creatinine and reported results.

## Abbreviations

ACE2: Angiotensin-converting enzyme 2
AKI: Acute kidney injury
COVID-19: Coronavirus disease 2019
ICU: Intensive care unit
KDIGO: Kidney disease improving global outcome
PEEP: Positive end expiratory pressure
SARS-COV-2: Severe acute respiratory coronavirus 2
