# Supplementary material for: Acute kidney injury in patients with SARS-CoV-2 infection
Source: Ann Intensive Care. 2020 Sep 3;10:117. doi: 10.1186/s13613-020-00734-z (PMC7471244; doi:10.1186/s13613-020-00734-z)
Supplement: Supplementary file 1 — Additional file 1: Figure S1. Boxplots depicting relationship between AKI stage 2 and 3 and C3 [ng/mL] (A), IL-6 [ng/mL] (B), sC5b9 [ng/mL] (C) and ferritin levels [mg/L] (D) levels, and predicted probabilities of severe AKI according to ferritin (E) and C3 (F) deciles (per log). Figure S2. Kaplan-Meier curve for day-28 survival in patients without AKI or with AKI stage 1 (n=63) compared to AKI stage 2 or 3 (n= 37). Figure S3. Kaplan-Meier curves for day-28 survival according to AKI (A and B) and AKI stages (C and D) in patients with (n=33, B and D) and without (n= 67, A and C) baseline creatinine value. Table S1. Factors associated with risk of AKI stage 2 and 3, compared to no AKI or AKI stage 1, after adjustment for modified SOFA and chronic kidney disease (logistic regression). Table S2. Interaction between missing baseline serum creatinine and reported results. [file 13613_2020_734_MOESM1_ESM.docx]

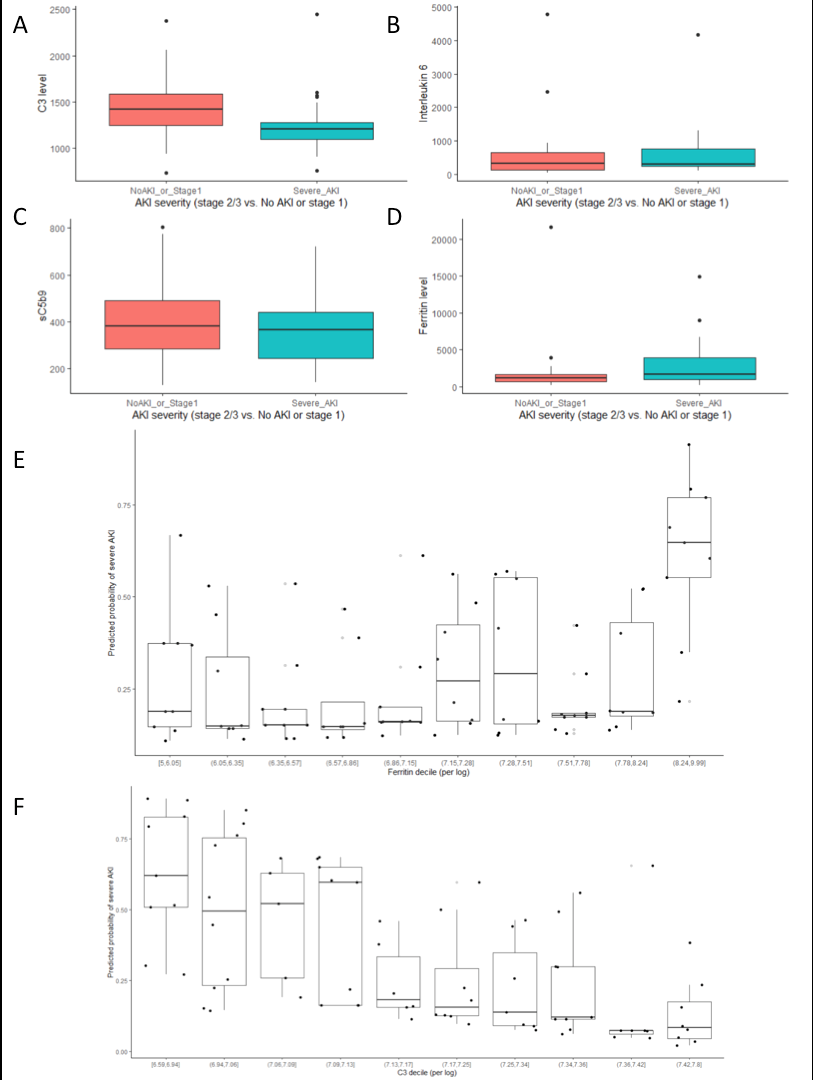


**Figure S1.** Boxplots depicting relationship between AKI stage 2 and 3 and C3 [ng/mL] (A), IL-6 [ng/mL] (B), sC5b9 [ng/mL] (C) and ferritin levels [mg/L] (D) levels, and predicted probabilities of severe AKI according to ferritin (E) and C3 (F) deciles (per log).


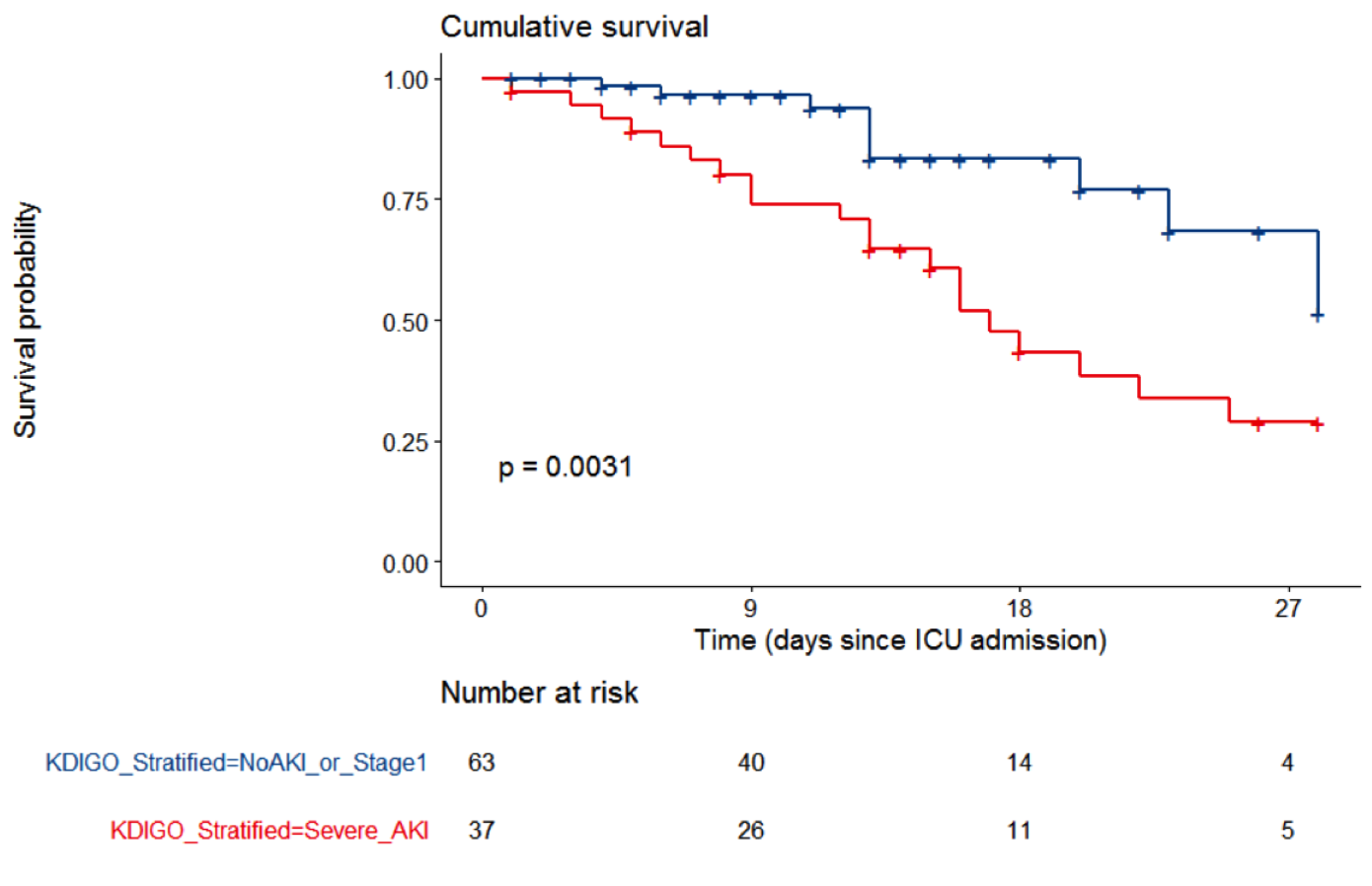


**Figure S2.** Kaplan-Meier curve for day-28 survival in patients without AKI or with AKI stage 1 (n=63) compared to AKI stage 2 or 3 (n= 37).


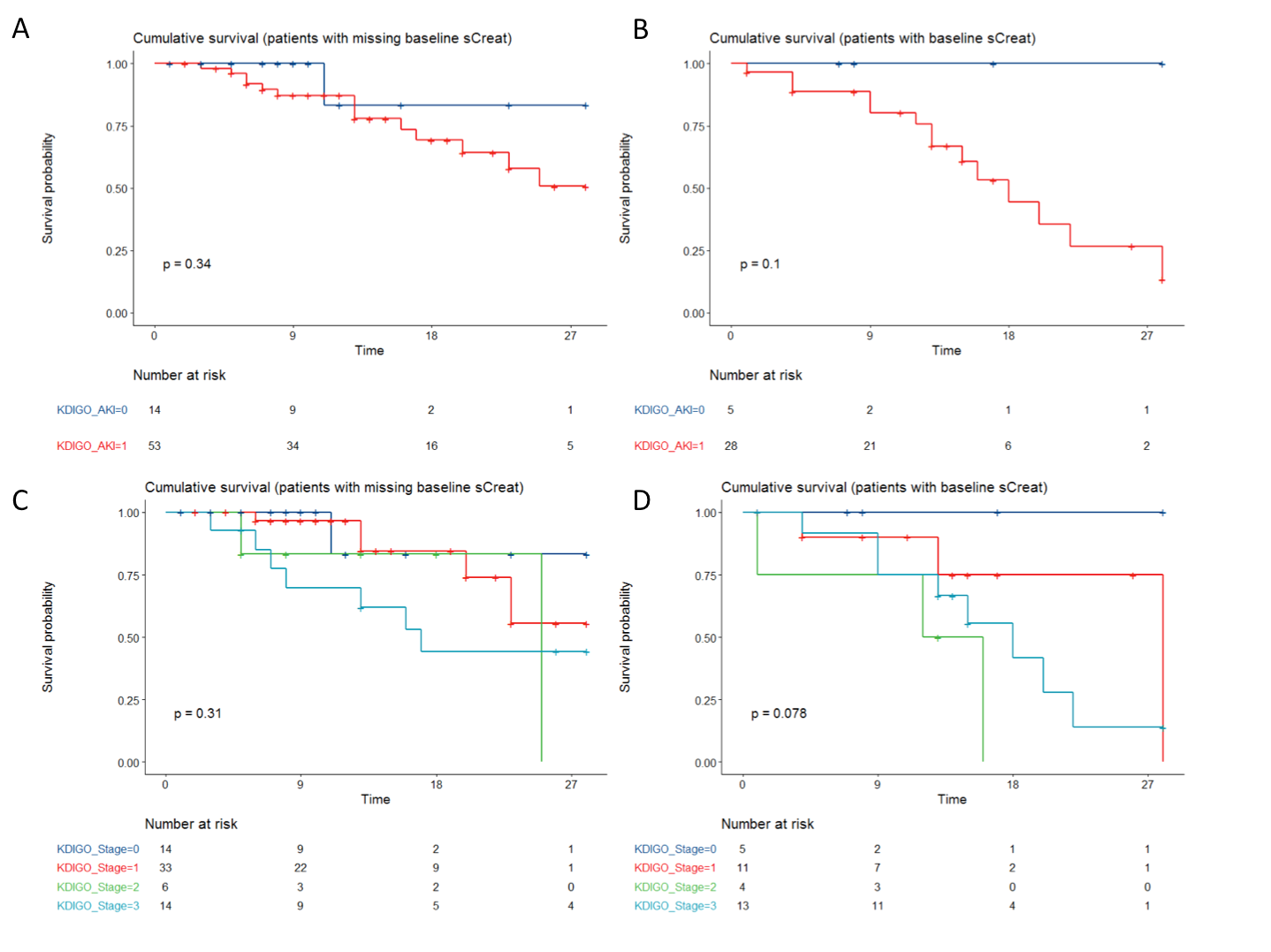


**Figure S3.** Kaplan-Meier curves for day-28 survival according to AKI (A and B) and AKI stages (C and D) in patients with (n=33, B and D) and without (n= 67, A and C) baseline creatinine value.

**Table S1**. Factors associated with risk of AKI stage 2 and 3, compared to no AKI or AKI stage 1, after adjustment for modified SOFA and chronic kidney disease (logistic regression)

|  | **Odds ratio** | **95%CI** | **P Value** |
| --- | --- | --- | --- |
|  |  |  |  |
|  |  |  |  |
| IL-6 at day 0 (per log increase) | 1.54 | 0.94-2.63 | 0.097 |
| Ferritin at day 0 (higher than median) | 2.64 | 0.94-7.94 | 0.072 |
| C3 at day 0 (higher than median) | 0.17 | 0.05-0.54 | 0.004 |
| sC5b9 at day 0 (per ng/mL) | 0.999 | 0.994-1.000 | 0.492 |
| PEEP at day 1 (per mmHg) | 0.993 | 0.884-1.11 | 0.906 |
|  |  |  |  |

**Table S2**. Interaction between missing baseline serum creatinine and reported results

| **Variable** | **Model** | **P-value for interaction** |
| --- | --- | --- |
|  |  |  |
|  |  |  |
| AKI | Mortality (univariate analysis) | 0.37 |
| AKI stage | Mortality (univariate analysis) | 0.96 |
| SOFA | AKI (logistic regression) | 0.10 |
| CKD | AKI (logistic regression) | 0.99 |
| IL-6 | AKI (logistic regression – Se analysis) | 0.23 |
| sC5b9 | AKI (logistic regression – Se analysis) | 0.09 |
| Ferritin | AKI (logistic regression – Se analysis) | 0.60 |
| PEEP at day 1 | AKI (logistic regression – Se analysis) | 0.30 |
| AKI | Mortality (Cox model) | 0.93 |
| SOFA | Mortality (Cox model) | 0.29 |
|  |  |  |
